# Genetic Determinants Enabling Medium-Dependent Adaptation to Nafcillin in Methicillin-Resistant Staphylococcus aureus

**DOI:** 10.1128/mSystems.00828-19

**Published:** 2020-03-31

**Authors:** Michael J. Salazar, Henrique Machado, Nicholas A. Dillon, Hannah Tsunemoto, Richard Szubin, Samira Dahesh, Joseph Pogliano, George Sakoulas, Bernhard O. Palsson, Victor Nizet, Adam M. Feist

**Affiliations:** a Department of Bioengineering, University of California San Diego, La Jolla, California, USA; b Collaborative to Halt Antibiotic-Resistant Microbes, Department of Pediatrics, University of California San Diego, La Jolla, California, USA; c Department of Biology, University of California San Diego, La Jolla, California, USA; d Skaggs School of Pharmacy and Pharmaceutical Sciences, University of California San Diego, La Jolla, California, USA; e Novo Nordisk Foundation Center for Biosustainability, Technical University of Denmark, Lyngby, Denmark; f Division of Biological Sciences, University of California San Diego, La Jolla, California, USA; Marquette University

**Keywords:** *Staphylococcus aureus*, antibiotic resistance, nafcillin, USA300, adaptive laboratory evolution, drug resistance mechanisms

## Abstract

Antimicrobial susceptibility testing standards driving clinical decision-making have centered around the use of cation-adjusted Mueller-Hinton broth (CA-MHB) as the medium with the notion of supporting bacterial growth, without consideration of recapitulating the *in vivo* environment. However, it is increasingly recognized that various medium conditions have tremendous influence on antimicrobial activity, which in turn may have major implications on the ability of *in vitro* susceptibility assays to predict antibiotic activity *in vivo.* To elucidate differential growth optimization and antibiotic resistance mechanisms, adaptive laboratory evolution was performed in the presence or absence of the antibiotic nafcillin with methicillin-resistant Staphylococcus aureus (MRSA) TCH1516 in either (i) CA-MHB, a traditional bacteriological nutritionally rich medium, or (ii) Roswell Park Memorial Institute (RPMI), a medium more reflective of the *in vivo* host environment. Medium adaptation analysis showed an increase in growth rate in RPMI, but not CA-MHB, with mutations in *apt*, adenine phosphoribosyltransferase, and the manganese transporter subunit, *mntA*, occurring reproducibly in parallel replicate evolutions. The medium-adapted strains showed no virulence attenuation. Continuous exposure of medium-adapted strains to increasing concentrations of nafcillin led to medium-specific evolutionary strategies. Key reproducibly occurring mutations were specific for nafcillin adaptation in each medium type and did not confer resistance in the other medium environment. Only the *vraRST* operon, a regulator of membrane- and cell wall-related genes, showed mutations in both CA-MHB- and RPMI-evolved strains. Collectively, these results demonstrate the medium-specific genetic adaptive responses of MRSA and establish adaptive laboratory evolution as a platform to study clinically relevant resistance mechanisms.

**IMPORTANCE** The ability of pathogens such as Staphylococcus aureus to evolve resistance to antibiotics used in the treatment of infections has been an important concern in the last decades. Resistant acquisition usually translates into treatment failure and puts patients at risk of unfavorable outcomes. Furthermore, the laboratory testing of antibiotic resistance does not account for the different environment the bacteria experiences within the human body, leading to results that do not translate into the clinic. In this study, we forced methicillin-resistant S. aureus to develop nafcillin resistance in two different environments, a laboratory environment and a physiologically more relevant environment. This allowed us to identify genetic changes that led to nafcillin resistance under both conditions. We concluded that not only does the environment dictate the evolutionary strategy of S. aureus to nafcillin but also that the evolutionary strategy is specific to that given environment.

## Abstract (precis)

The ability of pathogens such as Staphylococcus aureus to evolve resistance to antibiotics used in the treatment of infections has been an important concern in the last decades. Resistant acquisition usually translates into treatment failure and puts patients at risk of unfavorable outcomes. Furthermore, the laboratory testing of antibiotic resistance does not account for the different environment the bacteria experiences within the human body, leading to results that do not translate into the clinic. In this study, we forced methicillin-resistant S. aureus to develop nafcillin resistance in two different environments, a laboratory environment and a physiologically more relevant environment. This allowed us to identify genetic changes that led to nafcillin resistance under both conditions. We concluded that not only does the environment dictate the evolutionary strategy of S. aureus to nafcillin but also that the evolutionary strategy is specific to that given environment.

## INTRODUCTION

Staphylococcus aureus is a commensal Gram-positive bacteria that colonizes human skin, as well as nasal and respiratory tracts. Upon breaching skin or mucosal barriers, S. aureus can cause infections of skin, blood, and tissues (1). Although historically associated with hospital and health care infections, community-acquired methicillin-resistant S. aureus (CA-MRSA) infections are now widespread globally (2), of which USA300 is the most common clonal lineage in North America (3). MRSA TCH1516 is a well-studied representative USA300 strain isolated from an adolescent at the Texas Children’s Hospital in Houston with severe sepsis (4).

*In vitro* methods for evaluating antibiotic activity against bacterial pathogens were developed and standardized in 1961 as a “one size fits all” screen (5). This method has been paramount in antibiotic research, but translation to *in vivo* efficacy has been increasingly questioned (6, 7). Determination of the MIC for potential drugs has also varied considerably between “standard” testing media from different manufacturers (8) and with additionally supplemented cations (9). Differential susceptibility is even more pronounced between traditional testing media and more physiologically relevant medium conditions (i.e., taking factors such as supplemented cations, interaction with host factors, and nutrient availability into consideration) (10–12). In this study, differential antibiotic response was examined between standard bacteriological testing medium cation-adjusted Mueller-Hinton broth (CA-MHB) and Roswell Park Memorial Institute (RPMI) medium, a medium used in cell and tissue culture for mammalian cells, supplemented with 10% Luria-Delbruck (LB) (RPMI + 10%LB).

Despite the implications of medium-specific susceptibility of important pathogens, little work has been done to understand any medium-specific differential genetic response to tolerance under an antibiotic stress. Adaptive laboratory evolution (ALE) is an appropriate tool that can be utilized to meet this challenge and study the adaptive capabilities of microorganisms *in vitro*, as mutants that have differential resistance properties can be identified in a straightforward manner. ALE has been applied to study the adaptive response to a number of external stressors such as temperature (13, 14) or antibiotics (15–17). Specifically relevant to S. aureus, previous studies have utilized ALE to study the adaptive capabilities to various antibiotics (18–22). These studies have enabled an assessment of current and potential treatment strategies via identification of mutational targets and associated phenotypic changes that confer resistance to antibiotics of interests. Antistaphylococcal beta-lactams (e.g., nafcillin, oxacillin. flucloxacillin, cloxacillin) are the treatment of choice against serious methicillin-susceptible S. aureus (MSSA) infections (23, 24). A representative of this class, nafcillin, has been identified as one of the antibiotics with medium-dependent efficacies (11), making it an ideal candidate for this study. The Clinical and Laboratory Standards Institute (CLSI) breakpoint for nafcillin in CA-MHB is greater than or equal to 4 μg/ml, and susceptible is less than or equal to 2 μg/ml.

In this work, ALE was applied to uncover medium-specific mechanisms of resistance to nafcillin in a controlled setting. First, ALE was implemented to adapt S. aureus TCH1516 to both medium conditions (CA-MHB and RPMI + 10%LB), in order to optimize cellular performance and establish a suitable baseline with which to compare any further evolutionary work. Second, ALE was harnessed to study nafcillin resistance of such medium-adapted strains in order to gain insights into the genetic basis for adaptation in differing medium conditions. Finally, resistant strains were assessed for growth rate, effective nafcillin resistance, and virulence capabilities, so phenotypic trade-offs could be identified.

## RESULTS

### Laboratory evolution for adaptation to medium environments.

**FIG 1:**
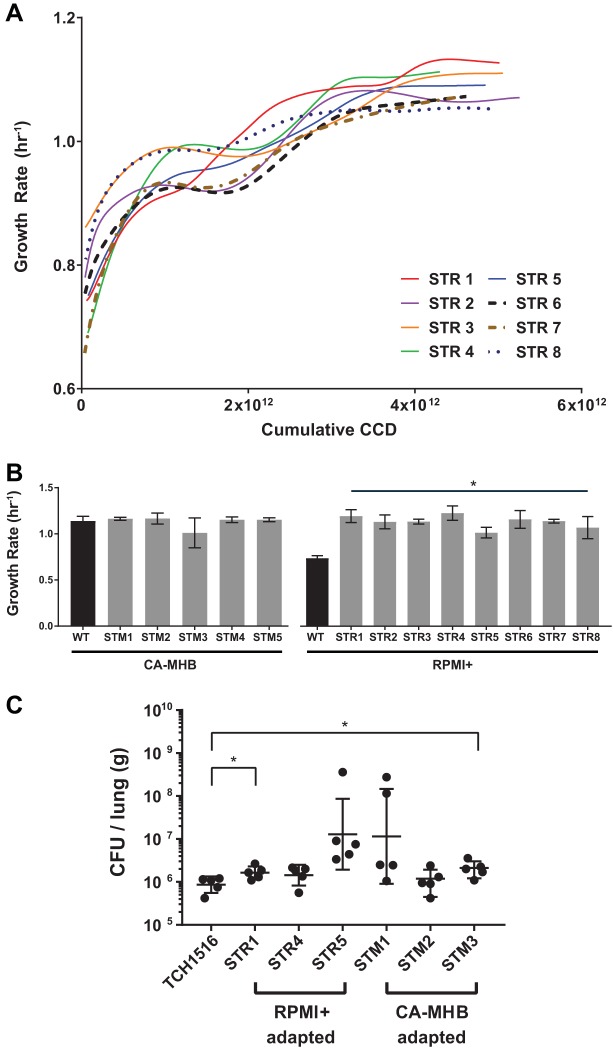
Medium adaptation of S. aureus TCH1516. (A) Fitness trajectories depicting growth rate increase throughout the course of the medium adaptation ALE in RPMI+. Strains STR 1, 4, and 5 served as progenitors for the medium-adapted starting points in the tolerance evolution. (B) Clonal growth rates for single clones isolated by streaking endpoint populations. Measurements were determined from biological duplicates and an average of two consecutive flasks. STR strains are S. aureus RPMI+-adapted strains. STM strains are S. aureus CA-MHB-adapted strains. Values that are significantly different (*P* < 0.0001) from the value for the wild type (WT) by two-way ANOVA are indicated by a bar and asterisk. (C) ALE-derived strains maintain parental lineage virulence in a murine pneumonia model of infection. Values that are significantly different (*P* < 0.05) by *t* test with Welch’s correction are indicated by a bar and asterisk.

S. aureus TCH1516 was forced to evolve under two medium conditions to understand how it adapts under growth rate selection to different nutritional environments. The two chosen medium types were CA-MHB and RPMI + 10%LB (referred to as RPMI+), since differential susceptibility to nafcillin was observed across both conditions (see Table S1 in the supplemental material) (11). Five independent populations of S. aureus TCH1516 were forced to evolve on CA-MHB, while eight independent populations were forced to evolve on RPMI+ for an average of 108 and 100 batch flask transfers, respectively (Table S2). Flask transfers were performed when an optical density at 600 nm (OD_600_) of 0.3 ± 0.02 or 0.434 g (dry weight [DW])/liter was achieved to prevent the cells from entering stationary phase, thus selecting for advantages in growth rate. Although no growth rate improvements were observed for evolutions performed in CA-MHB, population growth rates for S. aureus on RPMI+ increased from a starting wild-type growth rate of 0.75 ± 0.1 h^−1^ to 1.1 ± 0.1 h^−1^, an ∼1.5-fold increase, during a range of 4.52 × 10^12^ to 5.26 × 10^12^ cumulative cell cycle divisions (CCD) (Fig. 1A and B). CCD has previously been shown to effectively represent the time scale for ALEs in contrast to elapsed time or generations (25). It should be noted that the overall growth rate of the population at the end of the evolution on RPMI+ (1.06 ± 0.10 h^−1^) was similar to that of the starting growth rate on CA-MHB (1.12 ± 0.083 h h^−1^) (Table S2). Clonal isolates were selected from each of the final flasks of the independently evolved populations of the medium adaptation ALEs (i.e., endpoint clones) to RPMI+ (eight clones) and CA-MHB (five clones) in order to explore the phenotypes from the isolated evolved genotypes. Growth rates were measured for each of the endpoint clones, and there was concordance between the values observed for the populations at the end of the evolutions. The increase in growth rate of S. aureus TCH1516 through adaptation to RPMI+, but not to CA-MHB, was confirmed on the clonal level. Similar work has been performed forcing S. aureus to evolve in various medium conditions, although growth rates were not reported (26, 27). The identical growth rate between the two conditions evaluated here indicates an apparent maximum achievable growth rate for strain TCH1516 in a batch growth rich medium environment, given the stated evolution times. Following medium adaptation, the medium-adapted strains were evaluated for their virulence capabilities and sequenced to explore the genetic mechanisms behind observed fitness improvements.

10.1128/mSystems.00828-19.3 TABLE S1 MIC susceptibility testing of S. aureus TCH1516. Dagger indicates differential susceptibility greater than or equal to 4× across CA-MHB and RPMI + 10%LB. Download Table S1, DOCX file, 0.02 MB. Copyright © 2020 Salazar et al. 2020 Salazar et al. This content is distributed under the terms of the Creative Commons Attribution 4.0 International license.

10.1128/mSystems.00828-19.4 TABLE S2 Growth phenotypes for S. aureus TCH1516 populations that evolved on CA-MHB and RPMI+. Download Table S2, DOCX file, 0.02 MB. Copyright © 2020 Salazar et al. 2020 Salazar et al. This content is distributed under the terms of the Creative Commons Attribution 4.0 International license.

### Medium-adapted strain virulence in a murine model of pneumonia.

Continued passaging of pathogenic strains of bacteria *in vitro* can lead to attenuation, yielding derived laboratory strains that are disparate to those seen within patients (28). As multiple dedicated pathways are essential for virulence within a host, it is often the case that laboratory-evolved strains become nonpathogenic, due to the disruption of those pathways, leading to attenuation, therefore decreasing their clinical relevance. A murine pneumonia infection model was used to examine the virulence of medium-adapted strains in comparison to the pathogenic TCH1516 parental lineage. This model utilizes an intratracheal injection to establish a bacterial pneumonia and has been previously used to assess lung bacterial burdens (29). Surprisingly, despite RPMI+ serving as a better mimic for physiological conditions, strains that were adapted to RPMI+ did not have a virulence advantage within the host compared to those adapted to the standard laboratory growth medium (Fig. 1C). It was determined that medium-adapted strains maintain their pathogenicity and had no gross virulence defects in comparison with the TCH1516 parental lineage, indicating that these strains were not attenuated.

### Mutation analysis of whole-genome resequencing for medium adaptation.

Whole-genome sequencing was performed on evolved populations and selected clones from the ALE experiments on the two selected medium types to explore whether mutations could be linked to the observed fitness improvements. Sequences were analyzed to determine mutations from the multiple replicates under each condition (30, 31). For the CA-MHB condition, only endpoint clones were sequenced given the lack of an apparent fitness change during the course of the evolution, and a total of three unique mutations were found across all five replicates (two clones had no mutations detected [Table S3]). For the RPMI+ condition, there were 261 unique mutations across all of the intermediate and endpoint populations and clones selected during the experiment, with the clones having between 4 and 12 mutations each (Table S4). To focus the analysis, mutations were labeled as “key mutations” if a gene or genetic region contained multiple unique mutations across replicates or if an identical mutation appeared across independent ALE replicates. For RPMI+, there were eight genes or genetic regions that met these criteria, with three having greater than two instances. A summary of the RPMI+ medium adaptive mutations is shown in Table 1. For CA-MHB, there was no gene which shared mutations across two of the endpoint clones.

**TABLE 1:** Key reproducibly occurring mutations detected in the final populations and clones of S. aureus TCH1516 after adaptive laboratory evolution in RPMI+

Gene^*a*^	Specific function	Mutation type^*b*^	Protein and nucleotide change^*c*^	Strain(s)^*d*^
*apt*	Adenine phosphoribosyl transferase	SNP	D119E (GAT→GAA)	8
		SNP	H104Y (CAC→TAC)	1
		SNP	P76S (CCT→TCT)	6p
		SNP	G73D (GGC→GAC)	4
		SNP	A67V (GCT→GTT)	2p, 6, 7
		SNP	A67T (GCT→ACT)	5
		SNP	V66L (GTA→CTA)	1p
		SNP	V41L (GTA→TTA)	2

*mntA* (*znuC_1*)	Manganese ABC transporter	SNP	L11I (TTA→ATA)	4
		SNP	L2I (TTA→ATA)	2p
		SNP	M1M (TTG→ATG)†	3, 5, 6p, 7

*mntA*, *mntR* (*znuC_1*, *ideR*)	Manganese ABC transporter/Mn-dependent transcriptional regulator MntR	SNP	A→G, intergenic (−1/−121)	4
		SNP	C→T, intergenic (−2/−120)	2p
		INS	(GTTTAGGCTAACCTAATTAA)1→2, intergenic (−43/−79)	3, 5, 6p, 7

*stk1* (*prkC*)	Serine/threonine-protein kinase	SNP	A124P (GCG→CCG)	4
		SNP	V470D (GTT→GAT)	1

*cspA_2*	Cold shock protein CspA	SNP	A60V (GCT→GTT)	2
		DEL	Δ1 bp, coding (34/201 nt)	4

*dynA* (*RS07370*)	Bacterial dynamin-like protein	SNP	Q1098E (CAA→GAA)	2
		SNP	S618T (TCT→ACT)	6

*recJ*	Single-stranded-DNA-specific exonuclease RecJ	SNP	S757S (TCG→TCT)	3
		SNP	A348V (GCA→GTA)	8

*lyrA*	Lysostaphin resistance protein A	SNP	L48L (CTA→CTT)	1
		DEL	Δ1 bp, coding (1210/1260 nt)	5
		SUB	2 bp→AT, coding (1216 − 1217/1260 nt)	5

a The gene locus tag corresponds to USA300HOU_RSXXXXX. The gene nomenclature provided by prokka annotation, reflected in the mutation analysis, is shown in the parentheses.

b SNP, single nucleotide polymorphism; INS, insertion; DEL, deletion; SUB, substitution.

c nt, nucleotide; †, mutation led to formation of a start codon.

d p denotes population.

10.1128/mSystems.00828-19.5 TABLE S3 Mutations identified for S. aureus TCH1516 after medium adaptation to CA-MHB. Nomenclature example A1 F29 I1 R1 = ALE 1 Flask 29 Isolate (I1 = clone, I0 = population) Replicate 1. Download Table S3, XLSX file, 0.01 MB. Copyright © 2020 Salazar et al. 2020 Salazar et al. This content is distributed under the terms of the Creative Commons Attribution 4.0 International license.

10.1128/mSystems.00828-19.6 TABLE S4 Mutations identified for S. aureus TCH1516 after medium adaptation to RPMI+. Nomenclature example A1 F29 I1 R1 = ALE 1 Flask 29 Isolate (I1 = clone, I0 = population) Replicate 1. Download Table S4, XLSX file, 0.06 MB. Copyright © 2020 Salazar et al. 2020 Salazar et al. This content is distributed under the terms of the Creative Commons Attribution 4.0 International license.

In RPMI+ medium conditions, the most prevalent gene that mutated was *apt*, with all independent replicates containing at least one mutation in this gene, which remained present in the majority of endpoint clones (Table 1). The *apt* gene encodes an adenine phosphoribosyltransferase which enables nucleotide salvage reactions converting adenine to AMP (32). Mutations in this gene have also been discovered after *in vitro* passaging of S. aureus after exposure to increasing concentrations of vancomycin (18). Constructed *apt* deletion mutants experienced significant reduction in extracellular DNA (eDNA) release, a major constituent for biofilm stability and formation, low production of extra polymeric substances (33, 34), as well as increased resistance to Congo red (35).

An additional highly mutated region for growth rate optimization on RMPI+ was the *mntA* gene and its intergenic region upstream of both *mntA* and its regulator *mntR*. The *mntA* gene encodes a manganese permease subunit of an ATP binding transporter, while *mntR* encodes a metal-dependent transcriptional regulator (36). An identical mutation was identified in the start codon of *mntA* across three independent ALEs, modifying the initiation site from a suboptimal form (UUG) to AUG, which is the optimal start codon in prokaryotes (37, 38). Mutations in the intergenic region include two single nucleotide polymorphisms (SNPs) occurring 1 and 2 nucleotides upstream of *mntA*, likely affecting its promoter. The other intergenic change was an insertion of a 20-nucleotide sequence, 43 bp upstream of *mntA*. Acquisition of manganese is important for cell survival and replication of pathogens and is crucial for cell detoxification of reactive oxygen species (39). Inactivation of the MntABC transporter complex in another USA300 strain has been shown to attenuate virulence in *in vivo* mouse models (40). Manganese acquisition appears to be particularly relevant in endovascular infections. Disruption of *mntA*, *mntH*, *mntR*, or both *mntA* and *mntH* also significantly reduces intracellular survival in human endothelial cells. Bioavailable Mn is utilized by S. aureus to detoxify reactive oxygen species and protect against neutrophil killing, enhancing the ability to cause endocardial infections (41, 42).

Additional key mutations were identified in the RPMI+ growth rate adaptation: mutations in two genes encoding regulatory proteins, *cspA* and *stk1*, and in the *dynA*, *recJ*, and *lyrA* genes, encoding a GTPase, an exonuclease, and a protease, respectively (Table 1; see also Text S1 in the supplemental material).

10.1128/mSystems.00828-19.1 TEXT S1 RPMI+ medium adaptation additional mutations Download Text S1, DOCX file, 0.02 MB. Copyright © 2020 Salazar et al. 2020 Salazar et al. This content is distributed under the terms of the Creative Commons Attribution 4.0 International license.

The medium-adapted strains were subsequently used to understand S. aureus’ tolerization to nafcillin, with the goal of identifying the genetic basis of this process in the different medium environments.

### Laboratory evolution for adaptation to nafcillin tolerance.

A tolerance adaptive laboratory evolution (TALE) experiment was implemented to force medium-adapted strains of S. aureus TCH1516 to develop resistance to the β-lactam antibiotic nafcillin and identify mutations enabling an elevated growth rate under increasing antibiotic stress concentrations in both CA-MHB and RPMI+ medium environments. The S. aureus strains selected as starting strains of the TALE experiments consisted of the respective medium-adapted strains, denoted STM (CA-MHB) and STR (RPMI+). The starting strains for the TALE experiments were medium-adapted strains with distinct genotypes (Table 2).

**TABLE 2:** Tolerance phenotypes for S. aureus USA300_TCH1516 and medium-adapted evolved populations on CA-MHB and RPMI+^*a*^

Ancestor strain and strain	ALE no.	Initial growth rate (h^−1^)	Starting nafcillin concn (μg/ml)	Final growth rate (h^−1^)	Final nafcillin concn (μg/ml)	No. of flasks	CCD × 10^12^
RPMI+ TALE (SNFR)							
STR 1	7	1.17 ± 0.02	0.013	0.83 ± 0.12	65.52	184	15.2
	9	1.20 ± 0.03	0.013	0.83 ± 0.08	50.4	174	13.4

STR 4	13	1.23 ± 0.08	0.013	0.85 ± 0.09	83.16	191	14.5
	15	1.17 ± 0.08	0.013	0.83 ± 0.06	57.96	177	14.2
	17	1.04 ± 0.07	0.013	0.74 ± 0.11	65.52	172	13.6

STR 5	19	1.11 ± 0.1	0.013	0.87 ± 0.14	52.92	166	12.8
	21*	1.19 ± 0.04	0.013	0.99 ± 0.08	4.32	117	8.5
	23	1.22 ± 0.08	0.013	0.89 ± 0.14	57.96	171	13.6

CA-MHB TALE (SNFM)							
STM 1	7	0.79 ± 0.07	0.5	0.77 ± 0.17	61.2	72	3.94
	11	0.90 ± 0.12	0.5	0.87 ± 0.07	80.33	75	4.08

STM 2	13	0.94 ± 0.11	0.5	0.70 ± 0.03	61.2	68	3.75
	15	0.87 ± 0.14	0.5	0.76 ± 0.13	61.2	70	3.81

STM 3	19	0.97 ± 0.16	0.5	0.88 ± 0.08	61.2	74	3.95
	23	0.93 ± 0.11	0.5	0.93 ± 0.06	61.2	74	4.16

a Population growth rates for independent replicates were calculated by averaging the initial and final three flasks of the medium adaptation ALEs. An asterisk indicates premature end to experiment due to technical errors.

**FIG 2:**
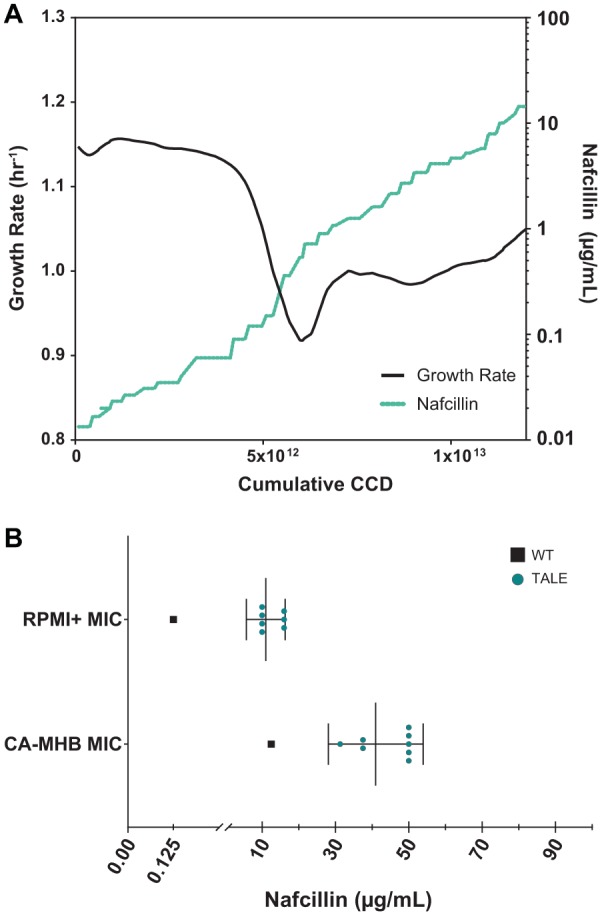
Nafcillin adaptation of medium-adapted strains derived from S. aureus TCH1516. (A) Fitness trajectory for a typical TALE experiment, showing population growth rate and continuously increasing antibiotic concentration. The selected trajectory depicts SNFR9 exposed to nafcillin in RPMI+. (B) A plot of the MICs for selected clones from endpoint populations after nafcillin tolerization. The MICs for the wild-type TCH1516 (black squares) and TALE strains (green circles) on the respective medium are shown.

TALE proved to be effective in developing strains with increased resistance to nafcillin in both RPMI+ and CA-MHB. Three medium-adapted starting strains per medium type (STM 1, 2, and 3 and STR 1, 4, and 5) were forced to evolve in duplicate or triplicate to generate a total of 14 independent evolutions (Table 2). Figure 2A details a typical TALE trajectory of the growth rate and the continuously increasing concentration of nafcillin in RPMI+ (Fig. S1 shows a CA-MHB nafcillin typical TALE trajectory). Over the course of evolution, S. aureus populations underwent an average of 3.93 × 10^12^ CCD and 72 flasks for CA-MHB and 13.81 × 10^12^ CCs and 175 flasks for RPMI+. Evolutions on RPMI+ were noticeably longer (Table 2) due to the differential susceptibility of S. aureus TCH1516 to nafcillin in the two medium conditions (11) (Table S1). The MIC on RPMI+ was ∼100-fold less compared to the MIC on CA-MHB for the respective starting strains (Table S5). The initial starting concentrations of nafcillin for the TALEs were therefore adjusted to ensure cell viability. Concentrations of nafcillin reached as high as 600× MIC_90_ on RPMI+ and 8× MIC_90_ on CA-MHB (Table 2) compared to the wild type on their respective medium.

10.1128/mSystems.00828-19.2 FIG S1 Fitness trajectory for a typical TALE experiment, showing population growth rate and continuously increasing antibiotic concentration. The selected trajectory depicts SNFM15 exposed to nafcillin in CA-MHB. Download FIG S1, EPS file, 0.1 MB. Copyright © 2020 Salazar et al. 2020 Salazar et al. This content is distributed under the terms of the Creative Commons Attribution 4.0 International license.

10.1128/mSystems.00828-19.7 TABLE S5 Nafcillin sensitivity of medium-adapted strains Download Table S5, DOCX file, 0.02 MB. Copyright © 2020 Salazar et al. 2020 Salazar et al. This content is distributed under the terms of the Creative Commons Attribution 4.0 International license.

Endpoint clonal isolates from each of the independent TALE replicates were selected to assess and confirm the increased nafcillin resistance phenotype. As expected, nafcillin resistance for the evolved clones was increased. However, the increase in tolerance observed for isolated clones did not quantitatively match the values tolerated by the TALE populations from which they were isolated (Fig. 2B). In RPMI+, an MIC_90_ ranging from 10 to 20 μg/ml was achieved for isolated clones compared to a range of 45 to 83 μg/ml observed in population endpoints. The same phenomenon was observed in a smaller degree for isolated clones from the TALE in CA-MHB. The MIC_90_ of nafcillin for CA-MHB TALE isolates ranged between 31.3 and 50 μg/ml compared to 61 to 87 μg/ml measured for TALE final evolved populations (Fig. 2B and Table 2). This can likely be attributed to population dynamics, kin selection (43), “bacterial cheating,” where overproduction of degradative enzymes can inactivate antibiotic molecules (44), or simply due to a difference in culturing methods under which the clonal MICs were determined compared to the culturing conditions during the TALE experiment (see Materials and Methods).

**FIG 3:**
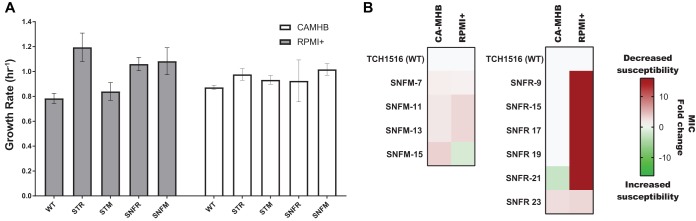
Phenotypic characterization of TALE strains. (A) Growth rates of wild-type, medium-adapted, and nafcillin-adapted strains. The graph shows the measured growth rates of several selected endpoint clones for strains derived from either RPMI+ (STR and SNFR) or CA-MHB (STM and SNFM) evolutionary conditions. White bars represent clonal growth rates in CA-MHB, and gray bars represent the growth rate for the same clones in RPMI+. The graph shows the growth rates of three tolerization endpoint clones from both medium conditions along a lineage. Data presented are averages from triplicates. A comprehensive ANOVA statistical analysis is provided in Table S5 in the supplemental material. (B) Heat map of the nafcillin MIC fold change of TALE strains compared to the wild-type MIC in both medium types. STM, S. aureus CA-MHB-adapted strain; STR, S. aureus RPMI+-adapted strain; SNFM, S. aureus nafcillin-adapted strain in CA-MHB; SNFR, S. aureus nafcillin-adapted strain in RPMI+.

To assess phenotypic trade-offs in the evolved strains, endpoint clonal growth rates were measured in their evolutionary medium as well as the alternate medium type utilized in this study (i.e., a medium swap) under no nafcillin stress. Characterizations were performed with both medium- and nafcillin-adapted clones. As shown above (Fig. 1B), RPMI+ medium-adapted strains (STR) saw a 52% increase in growth rate compared to the wild-type S. aureus TCH1516 (two-way analysis of variance [ANOVA], *P* < 0.0001) (Table S6). Medium adaptation to CA-MHB (STM) did not confer a fitness advantage in RPMI+ (two-way ANOVA, *P* = 0.8745). Strains with a higher resistance to nafcillin in RPMI+ (SNFR) resulted in a fitness tradeoff compared to medium-adapted strains in the same medium (STR) with an overall 11% decrease in growth rate (two-way ANOVA, *P* = 0.0054) (Fig. 3A and Table S6). For the medium swap conditions, there was an unexpected growth rate increase of 29% for strains evolved for resistance to nafcillin in CA-MHB (SNFM) when grown in RPMI+ compared to the progenitor strains that were adapted to CA-MHB (STM) (two-way ANOVA, *P* < 0.0001) (Fig. 3A and Table S6). There were no significant changes in growth rates observed across any of the strains analyzed in CA-MHB medium (Table S6).

10.1128/mSystems.00828-19.8 TABLE S6 Comparison of growth rates determined for clonal isolates, using two-way ANOVA analysis in Graphpad Prism 7.04. Download Table S6, DOCX file, 0.02 MB. Copyright © 2020 Salazar et al. 2020 Salazar et al. This content is distributed under the terms of the Creative Commons Attribution 4.0 International license.

### Mutational analysis for tolerance evolutions.

Whole-genome sequencing was performed on evolved populations and selected clones from TALEs on both medium types to determine shared or unique mutational mechanisms of nafcillin resistance phenotypes. Key mutations were again identified in a similar manner to those from the medium adaptation ALEs (i.e., if the gene or genetic region contained multiple unique mutations or the same mutation across independent ALE replicates). On average, there were fewer mutations in response to nafcillin stress on CA-MHB compared to RPMI+, as represented by the key mutations in the endpoint clones and populations (Tables 3 and 4). Endpoint clones and populations from evolution experiments on CA-MHB led to the identification of 13 unique key mutations across 5 genes, while the ones performed on RPMI+ presented 25 unique key mutations across 10 genes (Tables 3 and 4).

**TABLE 3:** Key mutations for final endpoint clones of S. aureus TCH1516 after tolerance adaptive laboratory evolution in CA-MHB to nafcillin (SNFM)

Gene^*a*^	Specific function	Mutation type	Protein and nucleotide change^*b*^	Strain
*apt*	Adenine phosphoribosyltransferase	SNP	G59D (GGC→GAC)	11
		SNP	I127N (ATT→AAT)	19
		SNP	K82E (AAA→GAA)	13

*pbuG*	Xanthine/guanine permease	SNP	Q6* (CAG→TAG)	7
		SNP	A84E (GCA→GAA)	23

*vraS*	Two-component sensor histidine kinase	SNP	G330D (GGT→GAT)	11
		SNP	T331I (ACA→ATA)	19

*vraT* (*RS10230*)	Transporter associated with VraSR	SNP	T8K (ACG→AAG)	13
		SNP	V199A (GTT→GCT)	23
		SNP	P126S (CCA→TCA)	19

*sgtB* (*mgt*)	Monofunctional transglycosylase	DEL	(T)7→6, coding (109/810 nt)	11
		SNP	Q215* (CAA→TAA)	15
		SNP	S121* (TCA→TAA)	13

a The gene nomenclature provided by prokka annotation, reflected in the mutation analysis, is shown in the parentheses.

b An asterisk indicates that a mutation led to a stop codon being formed.

**TABLE 4:** Key mutations for final endpoint clones of S. aureus TCH1516 after tolerance adaptive laboratory evolution in RPMI+ to nafcillin (SNFR)

Gene^*a*^	Specific function	Mutation	Protein and nucleotide change^*b*^	Strain(s)
*mecA*	Beta-lactam-inducible penicillin-binding protein	SNP	D586Y (GAT→TAT)	7, 9, 13, 15, 17, 23
		SNP	V488F (GTT→TTT)	19

*rpoD* (*sigA*)	DNA-directed RNA polymerase sigma subunit	SNP	A187T (GCA→ACA)	13
		SNP	A194V (GCA→GTA)	19

*gdpP*	Cyclic di-AMP phosphodiesterase	SNP	N182K (AAC→AAG)	9
		SNP	S222F (TCC→TTC)	23

*ywtF*	Putative transcriptional regulator	DEL	Coding (235/1218 nt)	7
		SNP	Y121* (TAC→TAG)	15
		SNP	D214N (GAC→AAC)	19

*codY*	CodY family transcriptional regulator	SNP	S204L (TCA**→**TTA)	9
		SNP	K205N (AAA→AAT)	15

*cdaA*	Cyclic di-AMP synthase	SNP	W76C (TGG→TGC)	7
		SNP	Q55H (CAG→CAT)	13
		SNP	A80S (GCT→TCT)	17

*ssaA2_4*	Staphylococcal secretory antigen	SNP	W70* (TGG→TAG)	9
		SNP	C45Y (TGT→TAT)	15
		SNP	G65V (GGC→GTC)	17

*oatA* (*oatA_2*)	*O*-Acetyltransferase	SNP	G451S (GGT→AGT)	7
		DEL	Coding (1239-1250/1812 nt)	9
		DEL	Coding (29/1812 nt)	13
		DEL	Coding (1327/1812 nt)	15
		SNP	E341* (GAA→TAA)	19

*vraS*	Sensor histidine kinase	SNP	G92V (GGC→GTC)	7
		SNP	V66L (GTA→CTA)	17, 19, 23

*RS08710*	Heme uptake protein MmpL11	DEL	Coding (2065-2067/2280 nt)	7, 19

a The gene nomenclature provided by prokka annotation, reflected in the mutation analysis, is shown in the parentheses. The gene locus tag corresponds to USA300HOU_RSXXXXX.

b An asterisk indicates that a mutation led to a stop codon being formed.

In CA-MHB, the majority of key mutations had been previously identified as being related to a resistance phenotype. One of the most frequently mutated gene sets were those that encoded the regulatory system VraSRT. In fact, *vraT* is a negative regulator of the *vraSR* operon which controls transcription of a number of genetic determinants involved in cell wall synthesis and cell division (45). Five of the 13 total key mutations under this condition were SNPs in the genes of this system. This regulatory system has also been shown to be mutated under vancomycin selection pressure in a different USA300 S. aureus strain, which also decreased daptomycin susceptibility (46). Another mutated gene was *apt*, which also occurred in RPMI+ medium adaptation ALE. This is interesting, as this might be the reason why SNFM clones presented an improved growth rate in RPMI+ conditions (Fig. 3A). As discussed earlier, *apt* enables nucleotide salvage reactions, a much more energetically favorable pathway than *de novo* nucleotide synthesis (47), and it has been implicated in the stringent response of bacteria to stressful conditions (32, 48). Mutations resulting in an amino acid substitution and a premature stop codon were discovered in *pbuG*, which encodes a guanine/xanthine permease. A Bacillus subtilis mutant with defects in *pbuG* displayed impaired uptake rates of nucleoside sugars guanine and hypoxanthine as well as resistance to toxic purine analog compounds (49). There has also been evidence to suggest a role between purine biosynthesis and increased resistance to vancomycin and daptomycin, two other membrane- and cell wall-targeting antibiotics (50, 51). The last key mutated gene that saw multiple mutations across TALE replicates in CA-MHB was *mgt*, or *sgtB* referred to elsewhere, whose gene product is a monofunctional glycosyltransferase responsible for elongation of the glycan strands using lipid-linked disaccharide-pentapeptide as the substrate (52). Each of the mutations in *sgtB* seems to lead to open reading frame disruption (Table 3), possibly abolishing its transcription. This glycosyltransferase is nonessential in S. aureus (53–56), but it seems to be upregulated upon treatment with cell wall-targeting antibiotics, including oxacillin (57). Furthermore, inactivation of *sgtB* in the USA300 S. aureus LAC strain has demonstrated increased resistance to several cell wall antibiotics (58).

In RPMI+, although there was a higher number of mutations, there was also a higher degree of parallelism, with 36% of key mutations compared to 25.5% in CA-MHB. The most targeted gene for mutation upon exposure to nafcillin in RPMI+ was *mecA* (Table 4). Mutations in *mecA* occurred in seven independent lineages, with an SNP at position 586 changing an aspartic acid residue to a tyrosine residue, comprising six of these mutations. Penicillin binding protein 2a (PBP2a) is encoded by *mecA* and is responsible for catalyzing transpeptidation of peptidoglycan during cell wall synthesis. The binding protein has long been thought to play a vital role in resistance to β-lactamase-resistant semisynthetic β-lactams (nafcillin, oxacillin, methicillin, etc.) due to its lower affinity for these antibiotics (59, 60). Emergence of S. aureus strains containing *mecA* has been hypothesized to be due in part to horizontal gene transfer from closely related staphylococcal species leading to formation of MRSA precursors (61). It has been discovered that PBP2a is essential for S. aureus survival, although it is able to replace transpeptidation activity by other PBPs, it still requires interaction with the transglycosylase activity of PBP2a (62).

Another highly mutated gene was *oatA*, which encodes an *O*-acetyltransferase. Genetic changes included formation of a premature stop codon, in-frame deletion of 12 bp, and two single base pair deletions. OatA encodes the enzyme required for O-acetylation of peptidoglycan by translocation of acetyl groups from a cytoplasmic source across the membrane (61). These results are consistent with previous data showing that exposure of MRSA to methicillin results not only in reduced peptidoglycan cross-linking but also in reduced peptidoglycan O-acetylation (63). O-acetylation is important for resisting autolysis activity from lysozymes (64) and has been shown to increase susceptibility to certain β-lactams (65). Reduction in O-acetylation has great implications for the host-pathogen relationship in S. aureus infections. Strains with mutations in O-acetyltransferase are more effectively killed by macrophages (66). Furthermore, S. aureus *oat* mutants have been shown to release more interleukin 1β (IL-1β) (66), a critical factor in rapid clearance of S. aureus bacteremia, as shown by the fact that patients with persistent bacteremia on antimicrobial therapy fail to mount a robust IL-1β response (67, 68). In fact, beta-lactam therapy has been shown to elicit a more robust IL-1β response compared to vancomycin therapy in patients with S. aureus bacteremia to potentially explain, at least in part, the more favorable clinical and microbiological data of beta-lactams over vancomycin (69). Coupled with previously cited phenotypic studies, our findings showing *oat* mutations induced by nafcillin selection pressure in physiological media on MRSA show direct evidence for a specific attenuation of virulence occurring at a genetic locus. These findings lend strong support of the role of nafcillin (and potentially other beta-lactams) as a potentially important adjunct therapy in MRSA bacteremia to enhance bacteremia clearance as previously reported (70).

Similar to what has been observed for the CA-MHB TALEs, the *vraSRT* system was also mutated, in this case mostly *vraS*, with a higher preference for amino acid position 66, where a valine was replaced by a leucine. As mentioned earlier, this operon controls transcription of a number of genetic determinants involved in cell wall synthesis and cell division (45).

Two genes involved in regulating levels of cyclic diadenosine monophosphate (c-di-AMP) inside the cell were also mutated: *gdpP*, encoding a phosphodiesterase, and *cdaA* (also known as *dacA*), which encodes an adenylate cyclase. Both DacA and GdpP are involved in nucleotide signaling pathways, while the former produces c-di-AMP, the latter degrades the cyclic dinucleotide molecule (71, 72). Studies suggest that SNP mutations in *dacA*, distinct from the ones presented here, affect methicillin resistance via nucleotide signaling by reducing c-di-AMP, resulting in faster growing, less resistant, and more virulent strains (73). On the other hand, SNP mutations in *gdpP* have been observed in S. aureus after repeated exposure to oxacillin concentrations of 200× MIC and insertional mutants revealed increased tolerance to both oxacillin and vancomycin, as well as altered phenotypic signatures (74, 75). Also, clinical isolates from patients with S. aureus lacking *mecA* determinants were shown to have mutations in *gdpP*, further implicating the phosphodiesterase in resistance to β-lactams (76). Other mutations included the following: *ywtF*, encoding a putative transcriptional regulator, belonging to a family of regulators associated with influencing virulence factors, antibiotic resistance, and cell envelope maintenance in various S. aureus species (77–79); *codY*, encoding a transcriptional regulator that acts as a repressor for more than 100 genes associated with branched-chain amino acid metabolism and virulence production under nutrient limiting conditions (80–82); *rpoD*, encoding a RNA polymerase subunit; *ssaA2_4*, encoding a secretory antigen precursor; and *RS08710*, encoding a heme uptake-related protein.

The overlap between mutations conferring resistance to nafcillin on the genetic level in both medium types was minimal. The key mutation overlap between the two TALE medium conditions was reduced to the *vraSTR* operon. As previously mentioned, mutations within this operon have previously been shown to increase expression of a cell wall stress stimulon leading to thicker cell wall and envelope (45, 83). From all of the additional key mutations observed in CA-MHB nafcillin tolerized strains, *pbuG* and *sgtB* also occurred once in RPMI+ nafcillin tolerized strains, in one replicate each (Table S7). On the other hand, from the other key mutations observed in nafcillin RPMI+ tolerized strains, only the *oatA* gene was mutated once in one CA-MHB replicate (Table S8). Interestingly, the genetic adaptation observed in the strains tolerized to nafcillin in RPMI+ did not translate to a resistant phenotype in CA-MHB (Fig. 3B), reiterating the medium-specific mechanisms employed toward nafcillin resistance. Nevertheless, the genes that were reproducibly mutated across the independent lineages under the CA-MHB and RPMI+ conditions have, for the most part, been previously identified as being associated with an antibiotic resistance phenotype.

10.1128/mSystems.00828-19.9 TABLE S7 Mutations identified for S. aureus TCH1516 during nafcillin tolerance in RPMI+. Nomenclature example A1 F29 I1 R1 = ALE 1 Flask 29 Isolate (I1 = clone, I0 = population) Replicate 1. Download Table S7, XLSX file, 0.04 MB. Copyright © 2020 Salazar et al. 2020 Salazar et al. This content is distributed under the terms of the Creative Commons Attribution 4.0 International license.

10.1128/mSystems.00828-19.10 TABLE S8 Mutations identified for S. aureus TCH1516 during nafcillin tolerance in CA-MHB. Nomenclature example A1 F29 I1 R1 = ALE 1 Flask 29 Isolate (I1 = clone, I0 = population) Replicate 1. Download Table S8, XLSX file, 0.03 MB. Copyright © 2020 Salazar et al. 2020 Salazar et al. This content is distributed under the terms of the Creative Commons Attribution 4.0 International license.

## DISCUSSION

For decades, methicillin-resistant S. aureus (MRSA) has been one of the major contributors to community- and hospital-acquired infections with a broad repertoire of infection type, severity, and human hosts (84). In the United States, this common commensal pathogen is responsible for more than 1 million cases of blood infection and close to 200,000 deaths (85). With such alarming figures, it becomes imperative to understand the underlying mechanisms of antibiotic resistance and adaptation to the host environment. Here, we present a method for determining differential mechanisms of resistance on the genetic level under different medium environments utilizing adaptive laboratory evolution, whole-genome sequencing, and phenotypic characterizations of evolved strains. Genotypes of generated strains were characterized to study fundamental underlying differences in how environmental considerations affect susceptibility at a systems level. Insights gained by analyzing repeatedly mutated regions across different medium conditions in tandem with phenotypic assessment can be leveraged to inform more effective treatment strategies and identify novel drug targets. Thus, the major findings from this work include the following: (i) a significant growth rate increase via genetic adaptation to physiological medium (RPMI+) compared to a negligible one observed in CAMHB; (ii) no gross virulence attenuation observed in medium-adapted strains in a pneumonia model of infection; (iii) medium-specific adaptation toward nafcillin tolerance, attributed to parallely mutated genes, mostly related to membrane and cell wall integrity; (iv) key mutated genes previously shown to be associated with clinical resistant strains; (v) mutations in genetic loci under nafcillin selection pressure that could allow for enhanced intracellular survival. These findings support this approach to better understand clinically relevant adaptive strategies of bacteria that may influence not just antibiotic resistance, but also host-pathogen interactions.

Adaptive laboratory evolution was successful in generating medium-adapted strains of S. aureus TCH1516 to a more physiologically relevant medium, RPMI+. Strains adapted to RPMI+ (STR) saw an increase in growth rate, while no such increase was observed in CA-MHB-adapted strains (STM) (Fig. 1B). Mutations identified in RPMI+-adapted strains showed a high degree of evolutionary parallelism with mutations in the *apt* and *mntA* genes occurring in almost all of the independent ALE replicates (Table 1). Both gene products have been associated with the SOS stringent response in stressful conditions, while *mntA* specifically plays a key role in metal acquisition infection when the host limits availability (32, 39, 48, 86). A recent transcriptome analysis has shown that S. aureus TCH1516 is under manganese starvation upon cultivation in RPMI+ (96), strengthening the argument of a transcription and translation optimization of the *mnt* operon in the RPMI+ medium-adapted strains. Interestingly, mutations in *apt*, which enables nucleotide salvage reactions, were also identified in tolerance evolution to nafcillin, particularly when evolved on CA-MHB (Table 3). Mutations identified in this phosphoribosyltransferase likely play a crucial role in the improved growth rate in RPMI+ in the presence of no antibiotic for medium- and nafcillin-adapted strains (Fig. 3A).

The tolerance adaptive laboratory evolution (TALE) method proved successful in the generation of S. aureus TCH1516 strains resistant to nafcillin 2.5- to 4-fold higher compared to the wild type in CA-MHB and 80- to 160-fold higher in RPMI+ (Table 2 and Fig. 2B) after continuous exponential growth in the presence of increasing concentrations of nafcillin. The overlap of shared mutations between nafcillin resistance in each medium type point to several previously studied targets for antibiotic resistance (45, 54, 83). Evolution of antibiotic resistance in the tissue culture medium RPMI supplemented with 10% LB appears to enrich for several other mutations, particularly in *mecA* and other non-*mecA* genetic determinants (e.g., *oatA* and *vraS*). Mutations in *mecA* were all located in the active site of PBP2a (87), suggesting an alteration in the target for nafcillin, and thus enabling transpeptidase activity to proceed. Mutations affecting synthesis and acquisition of branched-chain amino acids, as well as biosynthesis of peptidoglycan and its precursors potentially suggest a reorganization of metabolic activity more representative of host infection (71, 81, 88). Importantly, mutations in *oatA* have been previously shown to have significant impact on S. aureus interaction with the host, potentially allowing enhanced intracellular survival to escape from largely extracellularly acting antibiotics like beta-lactams.

In summary, this study describes several mutations involved in adaptation to medium and nafcillin and discusses their possible role in those processes. These hypotheses warrant further investigation into the molecular mechanisms involved in such genetic adaptations, via reintroduction of such mutations into a wild-type strain using targeted genetic engineering approaches (89, 90) or biochemistry elucidation of protein activities and interactions. This study outlines specific mutations that can be tested via these approaches and provides strong contextual evidence of their causality. Furthermore, with a strain-agnostic approach, one could understand if these mutations are strain-specific or general adaptation mechanisms employed by S. aureus (91).

## MATERIALS AND METHODS

### Adaptive laboratory evolution and tolerance evolution of S. aureus USA300_TCH1516.

The adaptive laboratory evolution (ALE) experiment was begun by streaking the wild-type S. aureus USA300_TCH1516 (taxid 451516) on LB agar plates. Colonies (five for CA-MHB and eight for RPMI+) were then selected and grown overnight at 37°C in the appropriate medium. Each individual flask served as the starting point for independent ALE experiments. An automated liquid handling platform (92) was used to serially propagate the growing cultures and monitor growth rates. Each batch culture was grown in 15 ml of the respective medium at 37°C and well aerated with magnetic stirrers at 1,800 rpm. When the optical density (OD) reached a value of 0.3, 150 μl was inoculated into the next flask, thus maintaining a continuous exponential growth. The automated system measured the OD at 600 nm (OD_600_) algorithmically on a Tecan Sunrise Absorbance Microplate reader. When the optical density reached a value of 0.3 (Tecan Sunrise plate reader equivalent to an OD_600_ of 1 on a traditional spectrophotometer with a 1-cm path length), 150 ul was inoculated into the next flask, thus maintaining a continuous exponential growth. The OD_600_ values were converted to cell dry weight (DW) concentrations using a previously determined OD_600_-dry cell weight relationship for S. aureus (1.0 OD_600_ = 0.434 g DW/liter). Last, frozen stocks were taken intermittently throughout the course of the evolution experiments in 50% (vol/vol) glycerol solution and stored at –80°C. Tolerance evolution was performed similarly to medium adaptation as described above with the addition of continuously increasing concentration of nafcillin. The TALE method was adapted from the method in reference 93.

Growth rate calculations were determined and filtered if *R*^2^ correlation was less than 0.98. Growth data were then smoothed to minimize noise following methods described in reference 94, by applying a three-median repeat smooth followed by convolution with a symmetrical kernel containing weights (1/4, 1/2, 1/4) and ended with final three-median smooth. Smoothed data were then fit to a piecewise cubic spline. The time scale of cumulative cell cycle divisions (CCD) was computed following methods outlined in reference 25.

### MIC determination.

Azithromycin (Fresenius Kabi), ceftazidime (Hospira), clindamycin (Pfizer), colistin (Xellia Pharmaceuticals ApS), daptomycin (Cubist Pharmaceuticals), linezolid (Pfizer), meropenem (Fresenius Kabi), and vancomycin (Mylan International) were purchased from a clinical pharmacy. Ampicillin, ciprofloxacin, gentamicin, and nafcillin were all purchased from Sigma-Aldrich. All drugs were resuspended in 1× Dulbecco’s phosphate-buffered saline (DPBS) (Corning).

The bacterial strains to be used in antibiotic susceptibility testing were first streaked on Luria agar plates from stocks stored at –80°C (in 20% glycerol and 80% Mueller-Hinton broth [MHB]) and grown stationary at 37°C overnight. Isolated colonies were picked from the plate and inoculated into 5 ml of either CA-MHB (MHB [Difco] amended with 20 mg/liter Ca^2+^ and 10 mg/liter Mg^2+^) or RPMI+ (phenol-free RPMI 1640 [Gibco] amended with 10% Luria-Delbruck [LB] [Criterion]) medium in a 14-ml Falcon polypropylene round-bottom snap cap tube (catalog no. 352059; Corning) and grown shaking at 100 rpm at 37°C overnight. The following day the overnight cultures were subcultured 1:50 in the desired medium and volume in either the 14-ml snap cap tubes and grown shaking at 100 rpm at 37°C until they reached mid-logarithmic phase (∼OD_600_ of 0.4). Unless otherwise noted, experiments were conducted in Costar flat-bottom 96 well plates (catalog no. 3370; Corning) with a final volume of 200 μl/well.

For the MIC experiments, the bacteria were cultured in the same medium throughout (CA-MHB or RPMI+) prior to the addition of antibiotics. The mid-logarithmic-phase cultures were diluted to approximately 5 × 10^5^ CFU (∼OD_600_ of 0.002), and 180 μl was added to each experimental well of the 96-well flat-bottom plate (catalog no. 3370; Costar). Either 20 μl of 1× DPBS or 20 μl of the desired 10× drug stock was added into each culture-containing well. The plates were then incubated shaking at 100 rpm at 37°C overnight. Bacterial growth, as determined by measuring the OD_600_ of each well, was determined by utilizing an Enspire Alpha multimode plate reader (PerkinElmer). To determine the MIC_90_, defined as the amount of drug required to inhibit ≥90% of the growth of the untreated controls, the density of each drug-treated well was compared to the density of the untreated control well.

### Mouse studies.

All animal experiments were conducted under veterinary supervision and approved by the University of California San Diego (UCSD) IACUC. Bacterial pneumonia was established as previously described (29). In brief, S. aureus strains were grown overnight in cation-adjusted Mueller-Hinton broth (CA-MHB) and then used to inoculate fresh CA-MHB the day of the infection. Cultures were grown to logarithmic phase (∼OD_600_ of 0.4), washed three times in 1× DPBS (Corning), and resuspended to a concentration of 2.5 × 10^9^ CFU/ml. Juvenile 8-week-old female C57Blk/6J mice were treated with 100 mg of ketamine (Koetis)/kg of body weight and 10 mg of xylazine (VetOne)/kg and then intratracheally infected with 40 μl of the infection culture to give each mouse a 1 × 10^8^ dose using an operating otoscope (Welch Allyn). Mice were allowed to recover on a sloped heating pad and then returned to their home cage. Mice were euthanized 24 h postinfection through CO_2_ exposure followed by cervical dislocation. All five lobes of the lung were removed, placed into a 2-ml sterile microtube (Sarstedt) with 1 ml of 1× DPBS and 1-mm silica beads (Biospec), and homogenized for three cycles with one cycle consisting of 1 min on a MagNA lyser (Roche) followed by 1 min on ice. Homogenized samples were then serially diluted and spot plated on Luria agar (Criterion) plates, and then grown overnight at 37°C for CFU enumeration.

### Whole-genome sequencing and identification of mutations.

A total of 162 samples, including population and clonal samples were submitted for sequencing. Genomic DNA was isolated using Nucleospin Tissue kit (Macherey-Nagel). The resequencing library was constructed from the isolated genomic DNA using Kapa HyperPlus kit (Roche) according to the manufacturer’s instructions. Then, the library was sequenced using a MiSeq reagent kit v3 (Illumina) in 600-cycle paired-end recipe on an MiSeq instrument (Illumina). Resequenced samples were then processed utilizing a modified script of the software breseq v.0.32.1 (30, 31) to map the genomes of the generated strains to the ancestral genome for identification of genetic mutations. All generated strains were mapped to S. aureus USA300_TCH1516 and reannotated using Prokka (95) (NCBI accession number GCA_000017085.1).

### Data availability.

Newly determined sequence data were deposited in the NCBI database under accession numbers SRX3480972 to SRX3480983 (STM), SRR10341521 to SRR10341525 (STM), SRX3482887 to SRX3482918 (STR), and SRR8552606 to SRR8552775 (SNFM and SNFR).
