# Supplementary material for: Genetic Determinants Enabling Medium-Dependent Adaptation to Nafcillin in Methicillin-Resistant Staphylococcus aureus
Source: mSystems. 2020 Mar 31;5(2):e00828-19. doi: 10.1128/mSystems.00828-19 (PMC7112963; doi:10.1128/mSystems.00828-19)
Supplement: TABLE S1 [file mSystems.00828-19-st001.docx]

| **Antibiotics** | **WT TCH1516 (CA-MHB)** | **WT TCH1516 (RPMI+)** |
| --- | --- | --- |
| **Nafcillin †** | 12.5 | 0.125 |
| **Ampicillin †** | >512 | >64 |
| **Azithromycin †** | 250 | 1 |
| **Ceftazidime †** | 256 | 32 |
| **Ciprofloxacin** | 0.5 | 1 |
| **Clindamycin †** | 64 | 16 |
| **Colistin †** | 500 | 64 |
| **Daptomycin †** | 4 | 1 |
| **Gentamicin** | 2 | 1 |
| **Linezolid** | 2 | 2 |
| **Meropenam †** | 32 | 8 |
| **Vancomycin** | 1 | 1 |
