# Supplementary material for: Genetic Determinants Enabling Medium-Dependent Adaptation to Nafcillin in Methicillin-Resistant Staphylococcus aureus
Source: mSystems. 2020 Mar 31;5(2):e00828-19. doi: 10.1128/mSystems.00828-19 (PMC7112963; doi:10.1128/mSystems.00828-19)
Supplement: TABLE S2 [file mSystems.00828-19-st002.docx]

| **Strain** | **Media Type** | **Replicate** | **Initial Growth Rate (h^-1^)^a^** | **Final Growth Rate (h^-1^)^a^** | **Total #  Flasks** | **Cumulative CCD x 10^12^** |
| --- | --- | --- | --- | --- | --- | --- |
| TCH1516 | CA-MHB | STM1 | 1.17  ± 0.04 | 1.12  ± 0.04 | 112 | 6.07 |
|  |  | STM2 | 1.26  ± 0.11 | 1.18  ± 0.06 | 110 | 5.56 |
|  |  | STM3 | 1.28  ± 0.16 | 1.1  ± 0.08 | 109 | 5.65 |
|  |  | STM4 | 1.07  ± 0.16 | 1.13  ± 0.09 | 108 | 5.74 |
|  |  | STM5 | 1.12  ± 0.29 | 1.07  ± 0.08 | 104 | 5.72 |
|  | RPMI+ | STR1 | 0.73  ± 0.09 | 1.03  ± 0.04 | 104 | 5.02 |
|  |  | STR2 | 0.78  ± 0.16 | 1.16  ± 0.01 | 106 | 5.26 |
|  |  | STR3 | 0.79  ± 0.06 | 1.11  ± 0.05 | 104 | 5.06 |
|  |  | STR4 | 0.72  ± 0.14 | 1.01  ± 0.04 | 97 | 5.00 |
|  |  | STR5 | 0.86  ± 0.2 | 0.97  ± 0.1 | 100 | 4.85 |
|  |  | STR6 | 0.71  ± 0.05 | 1.13  ± 0.12 | 98 | 4.62 |
|  |  | STR7 | 0.68  ± 0.08 | 1.02  ± 0.07 | 98 | 4.52 |
|  |  | STR8 | 0.76  ± 0.18 | 1.04  ± 0.11 | 98 | 5.00 |

a.) Population growth rates for independent replicates were calculated by averaging initial and final 3 flasks of the media adaptation ALEs.
