# Supplementary material for: Genetic Determinants Enabling Medium-Dependent Adaptation to Nafcillin in Methicillin-Resistant Staphylococcus aureus
Source: mSystems. 2020 Mar 31;5(2):e00828-19. doi: 10.1128/mSystems.00828-19 (PMC7112963; doi:10.1128/mSystems.00828-19)
Supplement: TEXT S1 [file mSystems.00828-19-s0001.docx]

**RPMI+ Media Adaptation Additional Mutations**

Directly downstream of *apt* in the genome, single nucleotide polymorphism (SNP) mutations were identified in *recJ* whose product is an exonuclease specific for single stranded DNA. One of the mutations appeared and got fixed early in the evolution (at approximately 20 transfers) (Table S6). The *recJ* encoded exonuclease has been implicated in homologous recombination and DNA repair through the methyl-directed mismatch repair pathway in *E. coli* [^1^](https://paperpile.com/c/USgFmF/qDJl1). Additional key mutations were identified in the media adaptation ALE experiments. Two intragenic and two intergenic mutations were identified in *cspA*, which encodes for RNA chaperone protein colloquially named cold shock protein A. The CspA protein is part of the larger cold shock protein family whose expression are induced by a wide range of stresses[^2–4^](https://paperpile.com/c/USgFmF/eO8iX+XlUmY+yzZ5d). More recently, Δ*cspA* mutants of *S. aureus* NCTC 8325 were constructed which were demonstrated to affect carbohydrate and ribonucleotide metabolism, as well as stress response and virulence gene expression, potentially making the protein a global regulator associated in bacterial adaptation to stressful environments[^5^](https://paperpile.com/c/USgFmF/QDPtv). The *stk1* gene, encoding for Ser/Thr protein kinase, contained two unique SNPs appearing in two end point strains. The kinase is the only Ser/Thr protein kinase encoded for in *S. aureus* and has been coined PrkC, PknB, and Stk1[^6–8^](https://paperpile.com/c/USgFmF/ei9sp+hEOn3+DHyea). The kinase has been implicated in various physiological roles and responses as a regulator of  cell wall metabolism, virulence control, and antibiotic resistance[^9–11^](https://paperpile.com/c/USgFmF/rp44p+Nu1v2+WQOe7)**.** PrkC has been characterized and postulated to contain an extracellular PASTA domain (penicillin-binding protein and serine/threonine kinase associated)[^12^](https://paperpile.com/c/USgFmF/XZL6L)**,** where S. aureus lacking the protein became susceptible to 𝛽-Lactam antibiotics[^13^](https://paperpile.com/c/USgFmF/C6TJh). Two unique mutations were identified in two independent ALEs in an uncharacterized GTPase gene, *dynA*, flanked by genes encoding for an exonuclease and postulated iron uptake membrane protein. The last key gene with 2 unique mutations identified, in the media adaptation to RPMI+ was in *lyrA*. The gene product for *lyrA* is Lysostaphin resistance protein A. Insertional mutants in this gene have been found to be deficient in persistor formation and survival in *S. aureus* USA500[^14^](https://paperpile.com/c/USgFmF/ZsTbh).

**References:**

1. Cooper DL, Lahue RS, Modrich P. Methyl-directed mismatch repair is bidirectional. *J Biol Chem* 1993; **268**: 11823–9.

2. Duval BD, Mathew A, Satola SW, Shafer WM. Altered growth, pigmentation, and antimicrobial susceptibility properties of *Staphylococcus aureus* due to loss of the major cold shock gene cspB. *Antimicrob Agents Chemother* 2010; **54**: 2283–90.

3. Graumann PL, Marahiel MA. Cold shock proteins CspB and CspC are major stationary-phase-induced proteins in *Bacillus subtilis*. *Arch Microbiol* 1999; **171**: 135–8.

4. Willimsky G, Bang H, Fischer G, Marahiel MA. Characterization of *cspB*, a *Bacillus subtilis* inducible cold shock gene affecting cell viability at low temperatures. *J Bacteriol* 1992; **174**: 6326–35.

5. Caballero CJ, Menendez-Gil P, Catalan-Moreno A, *et al.* The regulon of the RNA chaperone CspA and its auto-regulation in *Staphylococcus aureus*. *Nucleic Acids Res* 2018; **46**: 1345–61.

6. Ruggiero A, Squeglia F, Marasco D, Marchetti R, Molinaro A, Berisio R. X-ray structural studies of the entire extracellular region of the serine/threonine kinase PrkC from *Staphylococcus aureus*. *Biochem J* 2011; **435**: 33–41.

7. Donat S, Streker K, Schirmeister T, *et al.* Transcriptome and functional analysis of the eukaryotic-type ser/thr kinase PknB in *Staphylococcus aureus*. *J Bacteriol* 2009. Available at: http://jb.asm.org/content/early/2009/04/17/JB.00117-09.full.pdf[.](http://paperpile.com/b/USgFmF/hEOn3)

8. Lomas-Lopez R, Paracuellos P, Riberty M, Cozzone AJ, Duclos B. Several enzymes of the central metabolism are phosphorylated in *Staphylococcus aureus*. *FEMS Microbiol Lett* 2007; **272**: 35–42.

9. Beltramini AM, Mukhopadhyay CD, Pancholi V. Modulation of cell wall structure and antimicrobial susceptibility by a *Staphylococcus aureus* eukaryote-like serine/threonine kinase and phosphatase. *Infect Immun* 2009; **77**: 1406–16.

10. Burnside K, Rajagopal L. Aspects of eukaryotic-like signaling in Gram-positive cocci: a focus on virulence. *Future Microbiol* 2011; **6**: 747–61.

11. Cai X, Zheng W, Li Z. High-Throughput Screening Strategies for the Development of Anti-Virulence Inhibitors Against *Staphylococcus Aureus*. *Curr Med Chem* 2017. Available at: http://dx.doi.org/10.2174/0929867324666171121102829[.](http://paperpile.com/b/USgFmF/WQOe7)

12. Yeats C, Finn RD, Bateman A. The PASTA domain: a beta-lactam-binding domain. *Trends Biochem Sci* 2002; **27**: 438.

13. Vornhagen J, Burnside K, Whidbey C, Berry J, Qin X, Rajagopal L. Kinase Inhibitors that Increase the Sensitivity of Methicillin Resistant *Staphylococcus aureus* to β-Lactam Antibiotics. *Pathogens* 2015; **4**: 708–21.

14. Wang W, Chen J, Chen G, *et al.* Transposon Mutagenesis Identifies Novel Genes Associated with *Staphylococcus aureus* Persister Formation. *Front Microbiol* 2015; **6**: 1437.
