# Supplementary material for: Genetic Determinants Enabling Medium-Dependent Adaptation to Nafcillin in Methicillin-Resistant Staphylococcus aureus
Source: mSystems. 2020 Mar 31;5(2):e00828-19. doi: 10.1128/mSystems.00828-19 (PMC7112963; doi:10.1128/mSystems.00828-19)
Supplement: TABLE S5 [file mSystems.00828-19-st005.docx]

|  | **NAF MIC_90_ (µg/mL)** | | |  |
| --- | --- | --- | --- | --- |
|  | **CA-MHB** |  | **RPMI+** | |
| WT (TCH1516) | 25.6 – 12.8 |  | 0.125 – 0.0625 | |
| **CA-MHB adapted** |  |  |  | |
| STM 1 | 8 – 6.4 |  | 0.1 | |
| STM 2 | 16 |  | 0.2 | |
| STM 3 | 25.6 – 12.8 |  | 0.1 – 0.0625 | |
| **RPMI+ adapted** |  |  |  | |
| STR 1 | 32 |  | 0.5 | |
| STR 4 | 51.2 – 32 |  | 1 | |
| STR 5 | 25.6 |  | 0.5 – 0.2 | |
