# Supplementary material for: Genetic Determinants Enabling Medium-Dependent Adaptation to Nafcillin in Methicillin-Resistant Staphylococcus aureus
Source: mSystems. 2020 Mar 31;5(2):e00828-19. doi: 10.1128/mSystems.00828-19 (PMC7112963; doi:10.1128/mSystems.00828-19)
Supplement: TABLE S6 [file mSystems.00828-19-st006.docx]

| **Tukey's multiple comparisons test** | **Mean Diff.** | **95.00% CI of diff.** | **Significant?** | **Summary** | **Adjusted P Value** |
| --- | --- | --- | --- | --- | --- |
| **CAMHB** |  | | | | |
| WT vs. STR | -0.1041 | -0.2935 to 0.08541 | No | ns | 0.5473 |
| WT vs. STM | -0.0598 | -0.2493 to 0.1297 | No | ns | 0.9044 |
| WT vs. SNFR | -0.05196 | -0.2414 to 0.1375 | No | ns | 0.9406 |
| WT vs. SNFM | -0.1444 | -0.3305 to 0.04165 | No | ns | 0.2046 |
| STR vs. STM | 0.04427 | -0.05701 to 0.1455 | No | ns | 0.7423 |
| STR vs. SNFR | 0.05211 | -0.04917 to 0.1534 | No | ns | 0.6094 |
| STR vs. SNFM | -0.04035 | -0.1351 to 0.05439 | No | ns | 0.7602 |
| STM vs. SNFR | 0.007842 | -0.09344 to 0.1091 | No | ns | 0.9995 |
| STM vs. SNFM | -0.08461 | -0.1793 to 0.01012 | No | ns | 0.103 |
| SNFR vs. SNFM | -0.09245 | -0.1872 to 0.002283 | No | ns | 0.0593 |
| **RPMI +10 %LB** |  | | | | |
| WT vs. STR | -0.4108 | -0.5723 to -0.2492 | Yes | **** | <0.0001 |
| WT vs. STM | -0.05546 | -0.217 to 0.1061 | No | ns | 0.8745 |
| WT vs. SNFR | -0.276 | -0.4375 to -0.1144 | Yes | **** | <0.0001 |
| WT vs. SNFM | -0.2984 | -0.4562 to -0.1406 | Yes | **** | <0.0001 |
| STR vs. STM | 0.3553 | 0.2495 to 0.4611 | Yes | **** | <0.0001 |
| STR vs. SNFR | 0.1348 | 0.02901 to 0.2406 | Yes | ** | 0.0054 |
| STR vs. SNFM | 0.1124 | 0.0124 to 0.2123 | Yes | * | 0.0195 |
| STM vs. SNFR | -0.2205 | -0.3263 to -0.1147 | Yes | **** | <0.0001 |
| STM vs. SNFM | -0.2429 | -0.3429 to -0.143 | Yes | **** | <0.0001 |
| SNFR vs. SNFM | -0.02243 | -0.1224 to 0.07752 | No | ns | 0.9708 |
